# Placebo aiTBS attenuates suicidal ideation and frontopolar cortical perfusion in major depression

**DOI:** 10.1038/s41398-019-0377-x

**Published:** 2019-01-29

**Authors:** Chris Baeken, Guo-Rong Wu, Kees van Heeringen

**Affiliations:** 1 Department of Head and Skin, Faculty of Medicine and Health Sciences, Ghent University, Ghent, Belgium; 2 Department of Psychiatry, University Hospital (UZBrussel), Brussels, Belgium; 3 Ghent Experimental Psychiatry (GHEP) lab, Ghent University, Ghent, Belgium; 4 Key Laboratory of Cognition and Personality, Faculty of Psychology, Southwest University, Chongqing, China

## Abstract

The application of repetitive transcranial magnetic stimulation has been shown to rapidly decrease suicidal ideation in major depressive disorder (MDD). However, the neural working mechanisms behind this prompt attenuation of suicidal thoughts remains to be determined. Here, we examined how placebo-accelerated intermittent theta burst stimulation (aiTBS) may influence brain perfusion and suicidal thoughts using arterial spin labeling (ASL). In a randomized double-blind sham-controlled crossover trial, 45 MDD patients received aiTBS applied to the left dorsolateral prefrontal cortex (Trial registration: http://clinicaltrials.gov/show/NCT01832805). With each ASL scan measurement, suicidal ideation was assessed with the Beck Scale for Suicidal Ideation (BSI) and depression severity with the Beck Depression Inventory (BDI). Compared with active stimulation, the attenuation of suicidal ideation after 4 days of placebo aiTBS was related to significant frontopolar prefrontal perfusion decreases. These findings were unrelated to changes in depression severity scores. Although both active and sham aiTBS resulted in prompt decreases in suicidal ideation, specifically sham aiTBS significantly attenuated frontopolar perfusion in relation to reductions in BSI scores. Our findings show that in accelerated neurostimulation paradigms, placebo responses are related to perfusion decreases in brain areas associated with higher cognitive processes, resulting in suicidal ideation attenuation.

## Introduction

Over the last decades, repetitive transcranial magnetic stimulation (rTMS) has been successfully used to treat unipolar and non-psychotic major depressive disorder (MDD), particularly when combined with antidepressant medication^1,2^. To increase response and remission rates, intensified rTMS treatment paradigms have been introduced more recently^3,4^. Instead of spreading rTMS sessions over several weeks, a similar amount of sessions is applied within only a few days, usually involving several sessions per day. Such accelerated rTMS treatment protocols result not only in decreases in depressive symptoms but also in prompt decreases of suicidal ideation^5^. For instance, George et al.^5^ applied high-frequency rTMS (10 Hz) to the left prefrontal cortex delivering 6.000 pulses/session, three times daily for 3 days (total nine sessions; 54.000 stimuli). Inpatients, who were admitted because of suicidal ideation or a suicide attempt, were randomized to active or sham rTMS. Trained and blinded raters administered the Beck Suicidal Scale Inventory (SSI^6^) at baseline and at the end of each day during the 3 days of treatment. After the first day of stimulation, active and sham rTMS were associated with a 50% and a 25% reduction in SSI scores, respectively. After completion of the intensified protocol, almost all subjects showed substantial reductions in suicidal ideation. These findings demonstrate that, although active stimulation in the first day was superior to sham, at the end of the protocol suicidal ideation decreased comparably in both study groups, indicating some form of placebo response. Our recent randomized controlled trial of accelerated intermittent theta burst stimulation (aiTBS, involving bursts of high-frequency stimulation in a short amount of time, thus delivering the same amount of pulses as in lower frequency protocols) showed similar rapid decreases in suicidal ideation in MDD patients after both active and sham stimulation^7^. Importantly, the attenuation of suicidal ideation scores was not related to changes in the severity of depression symptoms, suggesting that aiTBS treatment reduces suicidal ideation independently of an improvement in depressive symptoms. These findings thus also suggest placebo effects of TMS on suicidal ideation, independently of its effects on the severity of depressive symptoms.

Placebo (and nocebo) effects are well known in medicine as they have been reported following many kinds of treatments^8,9^. A placebo may consist of any form of treatment or application that is used for its ameliorative effect on a symptom or disease, but that is ineffective or not specifically effective for the condition being treated^10^. A placebo thus is not an inert substance or intervention, but it is rather the administration of this substance or intervention within a set of sensory and social stimuli that inform the individual that a beneficial treatment is given^11^. The psychophysiological responses elicited by placebos can be very specific depending on the provided information, resulting in mind/body interactions that are guided by subjective factors (e.g., expectations, beliefs, meaning, and hope for improvement) and relational parameters^8^. Placebo effects of rTMS on depressive symptoms may appear large, but in fact are of a similar magnitude as those of psychopharmacotherapy^12^. In addition, a recent meta-analysis of rTMS studies in depressed patients showed that the magnitude of placebo response is associated to the effect size in the actively treated group, suggesting that the placebo response is a component of the therapeutic response to rTMS^13^.

Placebo responses (To avoid confusion on the terms “placebo,” “placebo effect,” and “placebo response”, here we will use the term placebo response, referring to the outcome caused by a placebo manipulation; reflecting the neurobiological and psychophysiological response to an inert substance or sham treatment and is mediated by various factors within the treatment context^14^.) are not limited to neuropsychiatric disorders, but they have been particularly documented in a variety of such disorders, including chronic pain, addiction, Alzheimer’s and Parkinson’s disease. Such responses are usually, but not unequivocally, associated with decreases of neural activity involved in brain regions^8,15^. In general, it has been suggested that placebo responses correlate with changes in a core network of brain regions associated with the default mode network (DMN) that is involved in self-generated emotion, self-evaluation, thinking about the future, social cognition, and valuation of rewards and punishment^16^. Placebo responses in antidepressant medication trials appear to correlate with functional changes in ventromedial prefrontal and in posterior midline structures (both part of the DMN) and in striatal regions^9,17^.

No studies have yet addressed placebo effects of non-invasive neurostimulation on brain perfusion and suicidal ideation in MDD patients. The current study therefore aimed at assessing suicidal ideation and correlating brain perfusion changes using arterial spin labeling (ASL) fMRI before and after neurostimulation treatment in a randomized sham-controlled trial in MDD patients. All patients were at least stage I treatment-resistant according to the Rush et al.^18^ criteria, meaning that they had at least one failed treatment trial with an SSRI or SNRI. ASL fMRI uses arterial water as an endogenous tracer to measure cerebral blood flow (CBF) and provides reliable absolute CBF quantifications^19,20^. At every ASL, fMRI measurement suicidal ideation and depressive symptoms were assessed using the 21-item Beck Scale for Suicidal Ideation (BSI^21^) and the 21-item Beck Depression Inventory (BDI-I^22^).

Although active and sham accelerated paradigms both have been shown to result in prompt attenuation of suicidal ideation, we hypothesized that perfusion pattern changes after sham aiTBS would be distinctly different compared with those after active aiTBS treatment. We expected brain perfusion reductions after sham aiTBS to be present particularly in brain areas related to higher cognitive processes, such as the DMN.

## Materials and methods

### Participants

The ethics committee of the Ghent University Hospital approved this monocentric randomized double-blind sham-controlled crossover study. The study was registered in the Clinical Trials.gov database (http://clinicaltrials.gov/show/NCT01832805). All subjects gave written informed consent. Inclusion criteria for the study were (1) a major depressive disorder selected with the Mini-International Neuropsychiatric Interview (MINI^23^), (2) right-handedness, (3) at least stage I treatment-resistance according to the Rush et al. criteria^18^, (4) at least 2 weeks free from psychotropic agents (except for the habitual use of benzodiazepines if necessary).

**Fig. 1:**
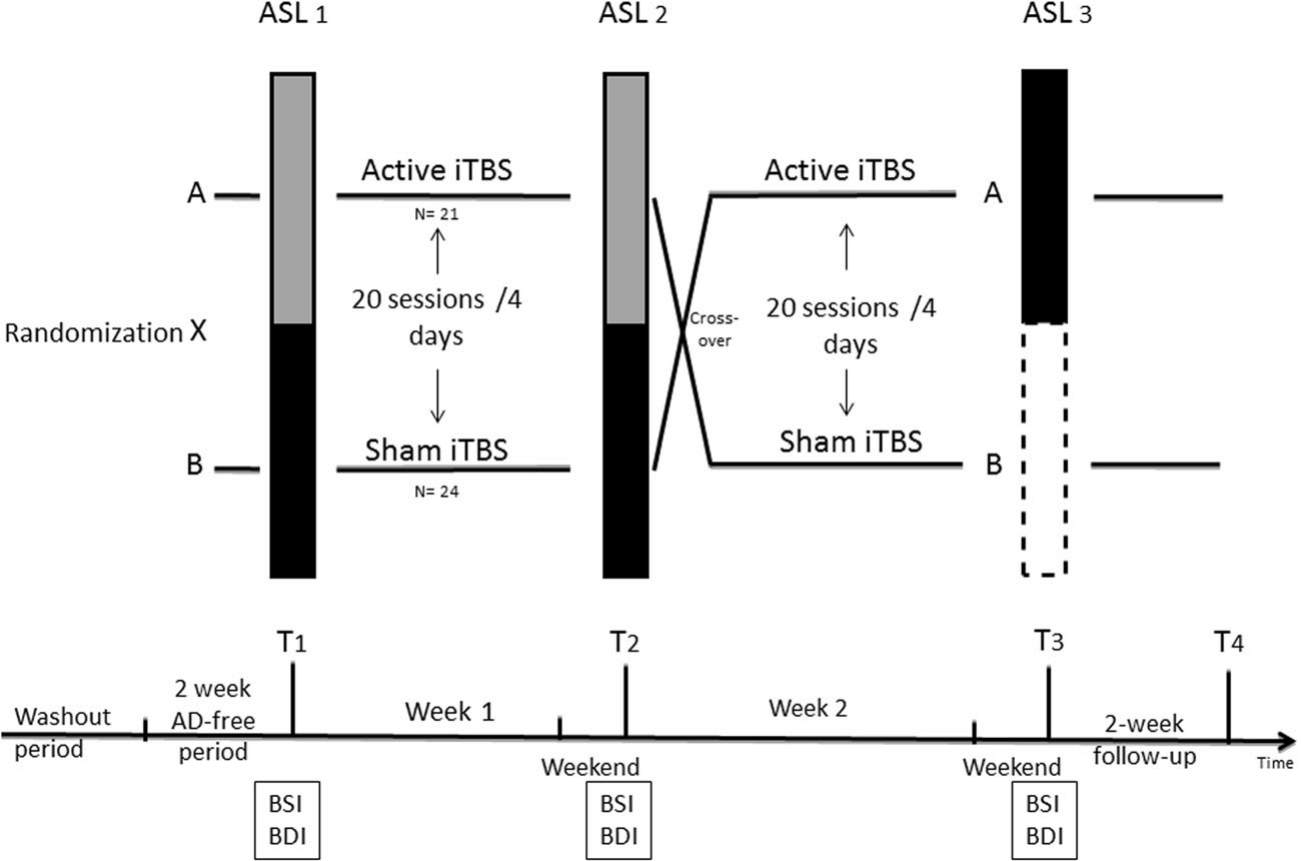
Flowchart of the experimental aiTBS treatment protocol. After a washout period, all TRD (treatment-resistant depressed) patients were at least 2 weeks antidepressant (AD) free before they underwent the first Arterial Spin Labeling (ASL_1_) scan at time T_1_ (on a Monday morning). Hereafter, patients were randomly divided into two groups to receive 20 sessions of real or sham aiTBS treatment, respectively. Line AB = a TRD patient who first received active aiTBS now receives sham; line BA = a patient who first received sham treatment now receives active aiTBS. This treatment was spread over the four succeeding afternoons (five daily sessions on Tuesday, Wednesday, Thursday, and Friday). In the second week, strictly the same treatment schedule was followed but with a change of stimulation. A second ASL scan was performed exactly 1 week after the first week (time T_2_) and a third ASL scan exactly after 2 weeks (time T_3_), always on a Monday morning. Before the start of every time point (T_1_, T_2_, and T_3_), suicide and depression severity symptoms were assessed with the 21-item Beck Scale for Suicidal Ideation (BSI) and the 21-item Beck Depression Inventory (BDI-I). Patients were clinically re-assessed after two weeks at T_4_ with the, however, without ASL scan. However, for this ASL study these delayed T_4_ measurements were not used as well as the last ASL _3_ scan at T_3_ in patients having received first active treatment, depicted in the dotted line white block

In total, 50 patients were included, and data on 45 patients (33 females) with the complete set of three ASL scans were included in the analyses (see Fig. 1). The full behavioral data were published in Duprat et al.^24,25^ and Desmyter et al.^7^, while functional connectivity findings were reported in Baeken et al.^26^.

### Clinical assessments

For the behavioral analyses, patients were assessed with the self-rating Beck Scale for Suicidal Ideation (BSI) and the Beck Depression Inventory (BDI-I) at each time point during the study (T_1_, T_2_, T_3_, and T_4_). The BDI assesses the severity of symptoms of depression in terms of how patients felt over the previous week, while the BSI measures the current intensity of suicidal ideations, intentions, and plans.

### aiTBS stimulation protocol

We applied accelerated intermittent TBS stimulation using a Magstim Rapid2 Plus1 magnetic stimulator (Magstim Company Limited, Wales, UK) with an active and a sham 70-mm Double Air Film figure-of-eight shaped cooled coil. The Magstim 70-mm Double Air Film sham coil is identical to its active variant, in that it is identical in all aspects but without stimulation output. By stimulating the peripheral nerves of the face and scalp, the Air Film sham coil looks, sounds, and feels the same as an active coil, both to the subject and operator but it does not deliver active stimulation of deep nerves (https://www.magstim.com/product/43/70mm-double-air-film-sham-coil). To accurately target the left DLPFC, we used Brainsight neuronavigation (Brainsight™, Rogue Resolutions, Inc) to locate the center part of the midprefrontal gyrus. The 20 iTBS sessions were spread over 4 days at five sessions per day, mounting a total of 32.400 stimuli (see Fig. 1 for a full overview, and Duprat et al.^24^ for more details). During each session, patients received 1620 pulses per session in 54 triplet bursts with a train duration of 2 s, and an intertrain interval of 8 s (from start to end and including the 2 s train duration). Between two sessions, there was a pause of ~15 min. Throughout the whole aiTBS treatment (active and sham), patients were blindfolded, fitted with earplugs, and were kept unaware of the type of stimulation they received. A stimulation intensity of 110% of the subject’s resting motor threshold (rMT) was maintained throughout the treatment.

### Brain imaging

The scans were performed on a Siemens 3-T TrioTim MRI scanner (Siemens, Erlangen, Germany) with a 32 channel SENSE head coil. To obtain individual anatomical information, all subjects underwent a first T1-weighted MRI (3D-TFE, TR/TE = 2530/2.58; flip angle = 7° FOV = 220 × 220 mm^2^; resolution = 0.9 × 0.9 × 0.9 mm³ number of slices = 176) of the brain. For every individual, we located the left DLPFC visually on the 3D surface rendering of the brain based on the known gyral morphology and marked the center part of the midprefrontal gyrus as the left DLPFC target (Brodmann 9/46).

Multi-delay pulsed arterial spin-labeled (pASL) images with a 3D GRASE readout were obtained with the following parameters: TR = 3.4 s, TE = 14.46 ms, labeling duration = 1400 ms, post-labeling delay changing from 300 to 3000 ms in steps of 300 ms, resulting in 12 pairs of slice-selective (SS) and non-selective (NS) images, scan duration = 5.26 min. This scanning procedure was repeated three times: ASL_1_ at baseline, ASL_2_ after the first week of aiTBS, ASL_3_ after the second week of aiTBS (see Fig. 1). During the ASL measurements, patients were asked to stay awake with their eyes closed.

### Data analysis

#### Behavioral data

All behavioral data were analyzed using SPSS 24 (IBM, Statistical Package for the Social Sciences, Chicago, IL, USA). The significance level was set at *p*<0.05, two-tailed, for all analyses.

#### Imaging data

The pASL and anatomical images were pre-processed and analyzed using FSL (FMRIB, Oxford, UK) and SPM12 (Wellcome Trust Centre for Neuroimaging, London, UK). The individual structural images were segmented into gray matter, white matter, and cerebrospinal fluid with SPM12. All NS and SS images were realigned to the mean image to correct for motion (using 4th-degree B-spline interpolation in SPM12). The mean image was affine-registered to the anatomical image, and the resulting warps were applied to the realigned images with SPM12. Then, 12 perfusion-weighted images were generated by surround subtraction, i.e., the differences between the paired SS and NS images. The perfusion-weighted images were submitted for CBF estimation using Oxford ASL (oxford_asl) tool in FSL. The partial volume effects in generated CBF maps were corrected by a regression algorithm in PETPVE12 toolbox^27^ (https://github.com/GGonEsc/petpve12). Finally, the generated CBF maps were spatially normalized into MNI space and smoothed with Gaussian kernel (8-mm full-width half-maximum).

First, to evaluate the influence of the initial severities of suicidal ideation on whole-brain perfusion, the baseline ASL maps (ASL_1_) were used in a multiple regression analysis, with the baseline ASL scans (ASL_1_), and the baseline BSI scores at T_1_ as the regressors, while correcting for age and gender. Because we were interested in the placebo effects of aiTBS on suicidal ideation, the significant surviving clusters were used as mask for the following regression analyses.

Second, to examine the relation between the change in perfusion and the change in BSI scores after the first week of aiTBS stimulation (T_1_–T_2_) separately for the group having received first active or sham treatment, we calculated the changes in perfusion between the baseline ASL scan and the second ASL scan, 3 days after the last aiTBS session (delta ASL = ASL_1_–ASL_2_). Gender, age, and the changes in depression severity (delta BDI = BDI_1_–BDI_2_) and suicidal ideation (delta BSI = BSI_1_–BSI_2_) were the covariates, while the change in perfusion (delta ASL = ASL_1_–ASL_2_) was the dependent variable. The change in depression severity was added as a co-variate because of the documented placebo responses in rTMS studies, including active stimulation^13^.

Third, to directly contrast the relation between the change in perfusion and the change in BSI scores between the group receiving sham versus active aiTBS in the first week (between subject analysis), we calculated the changes in perfusion between the baseline ASL scan and the second ASL scan, 3 days after the last aiTBS session (delta ASL = ASL_1_–ASL_2_). Gender, age, and the changes in depression severity (delta BDI = BDI_1_–BDI_2_) and suicidal ideation (delta BSI = BSI_1_–BSI_2_) were the covariates, while the change in perfusion (delta ASL = ASL_1_–ASL_2_) was the dependent variable.

Fourth, to examine the question whether the change in perfusion in relation to changes in BSI scores changed after the crossover from sham to active aiTBS, we also calculated the changes in perfusion between the second ASL scan and the third and last ASL scan (delta ASL = ASL_2_–ASL_3_) in the group of 24 patients receiving first sham aiTBS in the first week and active aiTBS in the second week (within subjects). Gender, age, and the changes in depression severity (delta BDI = BDI_2_–BDI_3_), and suicidal ideation (delta BSI = BSI_2_–BSI_3_) were the covariates. The change in perfusion (delta ASL = ASL_2_–ASL_3_) was the dependent variable.

For all ASL analyses, we applied the AlphaSim correction of *p*<0.05 as implemented in the REST toolbox 1.8 (restfmri.net/forum/), smoothness was estimated for all statistical maps, 1000 iterations). Corresponding brain regions were identified with the Talairach Daemon (search range: nearest gray matter) implemented in WFU PickAtlas^28^.

## Results

### Behavioral results

Twenty-one MDD patients received active iTBS treatment in the first week and sham stimulation in the second week. Twenty-four patients followed the reverse order (see Table 1). Given the intention-to-treat aiTBS protocol, we used a last-observation-carry-forward approach. There were no significant order differences in gender (*χ*^2^(45) = 0.16, *p* = 0.69), age (U = 211.500, n_1_ = 24, n_2_ = 21, *p* = 0.36), the number of comorbidities (U = 194.000, n_1_ = 24, n_2_ = 21, *p* = 0.17), baseline BSI (U = 246.500, n_1_ = 24, n_2_ = 21, *p* = 0.90), and baseline BDI (U = 224.500, n_1_ = 24, n_2_ = 21, *p* = 0.52). Baseline BDI and BSI scores correlated significantly (*r*_*s*_ = 0.31, *n* = 45, *p* = 0.04).

**Table 1:** Medians and interquartile ranges for the basic demographic quantities

Study sample	Baseline T1			T2			T3		
	All patients	First sham aiTBS	First active aiTBS	All patients	First sham aiTBS	First active aiTBS	All patients	First sham aiTBS	First active aiTBS
Gender (F:M ratio)	33:12	17:7	16:5						
Age	44.00 (19.00)	47.50 (20.75)	37.00 (18.50)						
Number of comorbidities	1 (1)	1 (2)	2 (1)						
BDI-I	29.00 (15.50)	31.00 (16.75)	29.00 (13.00)	26.00 (17.50)	25.50 (22.50)	27.00 (13.50)	24.00 (16.50)	20.00 (17.25)	24.00 (17.50)
BSI	10.00 (16.00)	11.00 (15.25)	6.00 (16.50)	2.00 (11.00)	3.00 (13.00)	1.00 (11.00)	1.00 (1.50)	1.00 (10.00)	0.00 (12.50)
Duration current depressive episode (years)	1.50 (3.83)	2.00 (4.00)	1.00 (3.25)						
Benzodiazepine dose (mg/day)	0.00 (5.00)	0.00 (8.75)	0.00 (2.50)						

Wilcoxon-paired *T* tests showed that 4 days of sham aiTBS resulted in a significant decrease in BSI scores (z = −2.24, n-ties = 19, *p* = 0.03), while 4 days of active aiTBS resulted in a borderline-significant decrease in BSI scores (z = −1.89, n-ties = 14, *p* = 0.06). The change in BSI scores (delta BSI week 1 = BSI_2_–BSI_1_) after 1 week of active stimulation (median = 0.00, IR = 7.50) was not significantly differed from the change after 1 week of sham aiTBS (median = 2.50, IR = 7.50; U = 224.500, n_1_ = 24, n_2_ = 21, *p* = 0.53). The change in BSI scores (delta BSI week 2 = BSI_3_–BSI_2_) during the second week after active stimulation (median = 0.00, IR = 1.50; z = −1.32, n-ties = 14, *p* = 0.19) was not significantly different from the change after sham stimulation (median = 0.00, IR = 3.75; z = −1.37, n-ties = 14, *p* = 0.17; U = 230.500, n_1_ = 24, n_2_ = 21, *p* = 0.62).

### ASL results

**Fig. 2:**
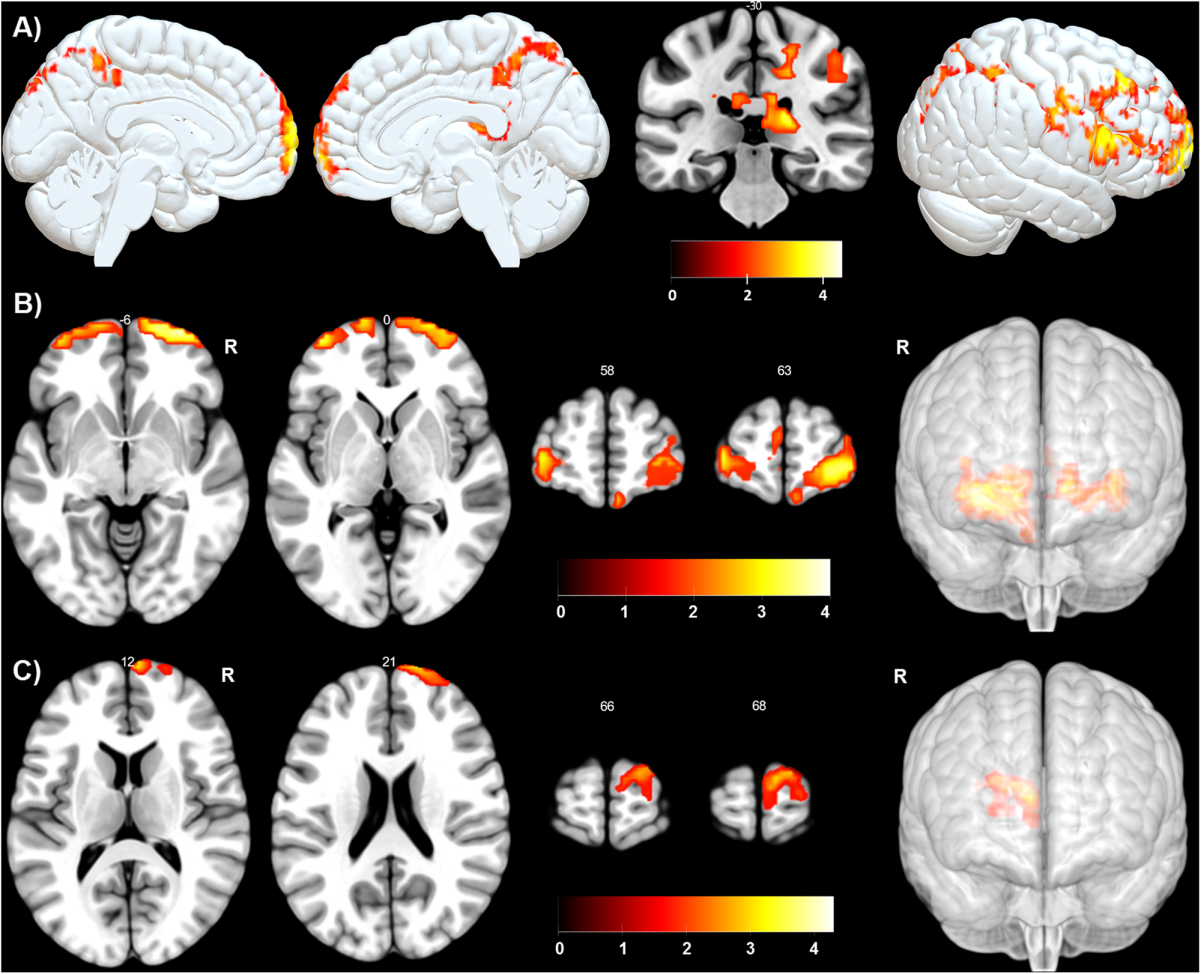
Sagittal, axial, and coronal views of the significant regression baseline ASL x BSI. See Table 2 for full details

First, the whole-brain regression analysis revealed two large positive regression clusters between baseline perfusion (ASL_1_) and the individual BSI (T_1_) scores in the superior frontal gyrus (Brodmann area (BA) 10: k = 1933 voxels: MNI coordinates: *x* = −6, *y* = 72, *z* = −3) and in the caudate (k = 1528 voxels: MNI coordinates: *x* = 12, *y* = −24, *z* = 24). This means that the higher the individual BSI scores at baseline the higher the perfusion in these areas. The minimum k was 850 voxels after AlphaSim correction at *p*<0.05; see Fig. 2a and Table 2a).

**Table 2:** Regression analyses

(A)	Cluster size (⌗voxels)	Anatomical region	Hemisphere	BA	*T*-value	df	Peak coordinates (*x*, *y*, *z*) (mm)
Baseline ASL_1_	BSI T_1_						
Positive	1933	Superior frontal gyrus	Left	10	4.46	1,41	−6, 72, –3
		Superior frontal gyrus	Right	10	4.34	1,41	24, 69, –3
		Orbitofrontal cortex	Right	11	4.15	1,41	24, 69, 0
		Superior frontal gyrus	Right	10	3.94	1,41	27, 66, –12
		Supramarginal gyrus	Right	40	3.26	1,41	42, –45, 57
		Dorsolateral prefrontal cortex	Right	9	3.31	1,41	60, 18, 27
	1528	Caudate	Right	–	3.76	1,41	12, –24, 24
		Inferior parietal lobule	Left	40	3.32	1,41	−33, –51, 33
		Supramarginal gyrus	Left	40	3.02	1,41	−45, –42, 45
		Somatosensory association cortex	Left	7	2.80	1,41	−24, –69, 33
		Precuneus	Right	–	2.65	1,41	12, –51, 57
Negative	No significant clusters emerged						
(B) Delta ASL (T_1_–T_2_)	Delta BSI (T_1_–T_2_)						
Active							
Positive	No significant clusters emerged						
Negative	No significant clusters emerged						
Sham							
Positive	258	Superior frontal gyrus	Right	10	3.87	1, 19	30, 66, –6
	162	Middle frontal gyrus	Left	10	3.63	1, 19	−39, 60, 0
Negative	No significant clusters emerged						
(C) Delta ASL (T_1_–T_2_)	Delta BSI (T_1_–T_2_)						
(between subjects)							
Active > sham							
Sham > active	111	Superior frontal gyrus	Right	10	4.29	1,38	6, 72, 15
(D) Delta ASL * (T_1_–T_2_) vs. (T_2_–T_3_)	Delta BSI (T_1_–T_2_) vs. (T_2_–T_3_)						
(within subjects)							
Active>sham	No significant clusters emerged						
Sham>active	No significant clusters emerged						

^*^Only those 24 TRD patients having received first sham aiTBS in the first week (T_1_–T_2_) before the crossover to real aiTBS treatment in the second week (T_2_–T_3_)

Second, the 24 TRD patients who received sham aiTBS in the first week (within subjects sham only; between T_1_ and T_2_) showed a significant positive association between the change in suicidal ideation (delta BSI = BSI_1_–BSI_2_) and the change in perfusion (delta ASL = ASL_1_–ASL_2_) in the bilateral frontal cortices (BA 10). This means that a decrease in perfusion after the first week of sham stimulation was associated with a decrease in suicidal ideation, while perfusion increases correlated with a worsening of suicidal ideation. The 21 TRD who received active aiTBS during the first week (within subjects active only; between T_1_ and T_2_) showed no significant positive or negative associations between perfusion changes (delta ASL = ASL_1_–ASL_2_) and BSI changes (delta BSI = BSI_1_–BSI_2_) (*p*<0.05, AlphaSim corrected; see Fig. 2b and Table 2b).

Third, compared with the group having had 1 week of active aiTBS treatment (*n* = 21), the group having received 1 week of placebo aiTBS (*n* = 24) (between subject analysis, active > sham iTBS) showed significant decreases in brain perfusion (delta ASL = ASL_1_–ASL_2_) in the superior frontal gyrus after placebo stimulation (BA 10: k = 111 voxels: MNI coordinates: *x* = 6, *y* = 72, *z* = 15) associated with significant decreases in suicidal ideation (delta BSI = BSI_1_–BSI_2_). The minimum k was 39 voxels after AlphaSim correction at *p*<0.05 (see Fig. 2b and Table 2c). This indicates that the improvement or worsening of suicidal ideation especially in the sham condition is mediated by these prefrontal cortices.

Fourth, focusing on the group of 24 patients receiving sham aiTBS in the first week and active aiTBS in the second week (within subjects; sham = > active crossover group, between T_2_ and T_3_; focussing on delta ASL = ASL_2_–ASL_3_ and delta BSI = BSI_2_–BSI_3_), showed no significant surviving regression clusters within the regions of interest (*p*<0.05, AlphaSim corrected). This suggests that the placebo effect on perfusion patterns is especially present in the first week (sham; T_1_–T_2_) and not any more in the second week of aiTBS treatment (active; T_2_–T_3_; see Table 2d.

## Discussion

This study shows that aiTBS treatment decreases suicidal ideation within 4 days of stimulation, whether active or sham. Our findings also indicate that suicidal ideation is primarily attenuated during the first week and not significantly further during the second week of aiTBS treatment. Remarkably, and as reported in Desmyter et al.^7^, this decrease in suicidal ideation lasts up to 1 month after baseline measurements (i.e., to the final behavioral assessment), also in patients who do not respond to this intervention in terms of a decrease in depressive symptoms. Brain imaging shows that higher baseline levels of suicidal ideation are associated with higher perfusion patterns in brain areas overlapping with the default mode network (DMN), particularly in the right hemisphere, and in the caudate. After 4 days of stimulation with sham, aiTBS perfusion decreases are found in the bilateral frontopolar cortices (BA 10), part of the DMN, and this is associated with a decrease in suicidal ideation. Importantly, these changes in suicidal ideation and perfusion are not related to a change in the severity of symptoms of depression.

Preceding a discussion of these findings in terms of their contribution to the understanding and prevention of suicide, a few methodological issues need to be addressed. Major advantages of the current study include the neuronavigated coil localization of the left DLPFC and the use of a sham 70-mm Double Air Film figure-of-eight shaped cooled coil that is identical to its active variant but without stimulation output. Nevertheless, all interpretations should be limited to antidepressant-free treatment-resistant depressed patients. Furthermore, we only examined one aspect of possible placebo aiTBS effects on the brain, namely cerebral perfusion. In addition, we can only conclude a decrease on suicidal ideation in a depressed but not highly suicidal group of patients, where suicide attempts within the last 6 months were an exclusion criterion. Lastly, although patients were naive to the iTBS procedure in their first week of stimulation (between T_1_ and T_2_), in the second week were only those patients who had received first sham treatment, in their second week of active aiTBS (between T_2_ and T_3_), carry-over effects or blinding issues may have interfered with the perfusion findings.

Given the fact that a number of findings are similar to those of previous studies, the impact of these limitations appears to be limited. Georges et al.^5^ showed that high-dose left prefrontal sham and active rTMS results in prompt decreases in suicidal ideation during a 3-day protocol. The imaging findings showing cerebral correlates of suicidal ideation are in keeping with those from previous studies of suicidal ideation and behavior. The ^99m^Tc HMPAO SPECT imaging study of Amen et al.^29^ showed significantly higher perfusion in the right hemisphere when suicidal depressed patients were compared with a non-suicidal depressed group. In vivo and post-mortem neurobiological studies point at the involvement of the DMN, ventromedial and parietal regions, and the caudate area in increased suicide risk^30–38^.

The involvement of the frontopolar cortex, commonly referred to as “Brodmann area 10”, is of particular interest. This part of the prefrontal cortex is proportionally larger in volume relative to the rest of the human brain^39^. The frontopolar cortex is involved in higher cognitive functions, especially in the integration of executive functions and cognitive flexibility. Impairments in cognitive flexibility are linked with suicidal ideation and behavior^40^. Post-mortem studies examining BA 10 of individuals who died by suicide show increased levels of proinflammatory cytokines in teenagers, reduced levels of BDNF in female suicides, and increased levels of neuropeptides, including CRH in male and female suicides^35^. Neurostimulation techniques, such as electroconvulsive therapy (also known for its prompt anti-suicidal effects) and deep brain stimulation targeting the nucleus accumbens, have also been found to result in frontopolar glucose metabolism decreases in treatment-resistant depressed patients^41,42^. Brain lesions, located in the ventromedial parts, are associated with markedly low levels of depression and lower scores on cognitive/affective symptoms of depression (such as guilt, self-dislike, and sadness), and possibly suicidal ideation^43^. While beneficial placebo effects of antidepressant pharmacotherapy on depressive symptoms are well established^12^, such effects on suicidal ideation are much less clear. A large clinical study among depressed patients, comparing psychotherapy and medication treatments to placebo, showed no differences after adjustment for change in symptoms of depression^44^. Diminished severity of depression may thus drive reductions in suicidal ideation during antidepressant treatment, whether active or placebo. To study the placebo effect of aiTBS, the change in severity of depression was therefore added as a co-variate in the brain perfusion analyses in the current study.

The reduced frontopolar perfusion after sham aiTBS after 4 days of stimulation in the current study is in line with placebo-induced brain responses found in other pathologies, i.e., reduced pain-related brain activations during placebo analgesia, often correlated with psychophysical pain measures^9,15^. The involvement of BA 10 in response to painful events suggests that this frontopolar cortical area plays a critical role in the collation, integration, and high-level processing of nociception and pain^45^. As reductions in BA 10 perfusion were coupled with decreases in suicidal ideation in the current study, these findings may indicate changes in higher cognitive processes involving the formation of expectations^46^, but also self-referential thinking and appraisal, such as value processing^16^. Here, “value” is defined as an appraisal of the gain or cost (economic, social, or physical) for current and future well-being, made about the self and in consideration of one’s goals. Frontopolar perfusion decreases after sham aiTBS suggest that MDD patients re-appraise their current situation more positively and consequently feel less suicidal. Of interest, the stimulated area (the left DLPFC) has also repeatedly been shown to be involved in the processing of placebo effects^9^. Indeed, the major direct synaptic projections to the frontopolar cortex originate in higher-order association areas, such as the DLPFC^47,48^. The DLPFC may also be involved in maintaining and updating expectancies that drive the placebo effect by modulating cortico-subcortical and corticocortical pathways^49^. On the other hand, sham-induced placebo effects on perfusion patterns as seen after the initial sham aiTBS were not observed after the crossover from active to sham aiTBS treatment. This may be due to carry-over effects, but also suggests that placebo responses to accelerated rTMS paradigms already occur at an early stage in this treatment protocol.

The lack of significant brain perfusion alterations in relation to suicidal ideation changes after active aiTBS is surprising. This could suggest a lack of effect of active aiTBS on suicidal ideation, but it could also indicate that the underlying neurobiological mechanisms of action in promptly reducing negative thinking patterns after active neurostimulation could be quite different from mechanisms involved in sham responses. This latter interpretation is supported by findings from a resting state fMRI study in the same sample as used in the current study: the decrease in the severity of symptoms of depression and the levels of hopelessness after active aiTBS, but not after sham stimulation, was associated with stronger functional connectivity between the subgenual anterior cingulate cortex and the medial orbitofrontal cortex. These cortical areas both are involved in cognitive inhibition and depressive rumination^26^.

In conclusion, the current study findings indicate that sham aiTBS rapidly attenuates suicidal ideation in treatment-resistant depressed patients, while significantly reducing brain perfusion in the frontopolar cortex. Sham aiTBS may lead to a more positive appraisal of the current situation and thus to a reduction in suicidal ideation. It remains to be demonstrated whether or not these placebo effects are limited to accelerated rTMS paradigms. Future rTMS studies in depression should consider targeting the frontopolar cortical regions more directly, especially when suicidal ideation is present.
